# Inducer microRNAs in the glioma development: a concise review of mechanisms and insights into targeted therapy

**DOI:** 10.1186/s43046-025-00308-9

**Published:** 2025-08-18

**Authors:** Mahya Pourrahimi, Marjan Hesari, Hannaneh Houshmandpour, Omid Mirzaee, Hamed Fouladseresht, Ensiye Torki, Hosein Kouchaki, Reza Tabrizi, Abdolmajid Ghasemian, Silvia Barbaresi

**Affiliations:** 1 Department of Medical Sciences, Faculty of Medicine, Tehran Medical Sciences, Islamic Azad University, Tehran, Iran; 2 Biomedicine Research Facility II, University of San Diego School of Medicine, San Diego, USA; 3 Shiraz University of Medical Sciences, Shiraz, Iran; 4 Department of Immunology, School of Medicine, Isfahan University of Medical Sciences, Isfahan, Iran; 5 USERN Office, Fasa University of Medical Sciences, Fasa, Iran; 6 Clinical Research Development Unit, Valiasr Hospital, Fasa University of Medical Sciences, Fasa, Iran; 7 Noncommunicable Diseases Research Center, Fasa University of Medical Sciences, Fasa, Iran; 8 Department of Movement and Sports Sciences, Ghent University, Ghent, Belgium

**Keywords:** MicroRNAs, Glioma, Diagnosis, Cancer therapy, Biomarkers

## Abstract

Gliomas represent predominant and fatal central nervous system (CNS) cancers lacking a gold standard of treatment, which need accurate prognosis, diagnosis, and intervention. Glioma accurate therapy using common traditional approaches such as surgical treatment, radiotherapy, and chemotherapy results insufficient mainly due to side effects, recurrence, and resistance. Scientific and medical challenges can be decreased considering novel therapeutic targets. The multiple and diverse role of microRNAs (miRNAs) in cellular processes has been demonstrated. The appreciation of miRNAs regulatory roles in cancer cell proliferation or growth inhibition opens new perspectives in the development of novel strategies targeting cancers. Six inducers (miRNAs) including miR-363-3P, miR720, miR-484, miR-890, miR-496, and miR-939-5p can develop into glioma cells with the potential of therapeutic targets. Therefore, the tracking of glioma stage and response to anticancer therapy is associated with various miRNAs. The objective of this review is to provide a comprehensive assessment of the role of miRNAs in glioma cancer development.

## Introduction

As predominant malignant tumors, gliomas occur in the central nervous system (CNS) and represent a heterogeneous group of tumors, which differ in their cellular origin, genetic alterations, and clinical behavior [1, 2]. As per the classification of the World Health Organization (WHO) for CNS tumors, each glioma is divided into different subtypes based on their histology and molecular features. The most common subtypes are diffuse or anaplastic astrocytoma, glioblastoma multiforme (GBM), anaplastic oligoastrocytoma, oligoastrocytoma, and anaplastic oligodendroglioma or oligodendroglioma [1, 3]. GBM, also known as grade IV astrocytoma, is the most malignant subtype of glioma and has a prevalence of approximately 80% of all gliomas and 48% of all primary malignant CNS tumors [4–6]. Despite advances in treatment strategies, such as surgical resection, radiation therapy, chemotherapy, and targeted therapy, the efficient therapy outcome among patients with GBM remains unclear, with a median survival time of less than 15 months [7, 8]. Recent findings have unraveled that the immune system plays a critical role in the development and progression of cancer, including GBM [7].

MicroRNAs (miRNAs) include short-chain non-coding RNA nucleotide sequences with substantial gene expression and cellular processes regulatory roles such as tumorigenesis. Dysregulation of miRNAs has been demonstrated in several cancers, such as gliomas. Therefore, understanding the role of miRNAs in GBM is essential for developing effective diagnostic and therapeutic strategies [9, 10]. In this review study, we provide an overview of the global miRNA mechanisms associated with tumorigenesis in patients with GBM. We focused on the role of miRNAs in various aspects of GBM pathogenesis, including glioma stem cell maintenance, tumor angiogenesis, invasion and metastasis, and immune evasion. We also discuss the potential of miRNAs as diagnostic and prognostic biomarkers and therapeutic targets in GBM. By elucidating the role of miRNAs in GBM development and progression, we aim to provide insights into potential novel therapeutic approaches for this devastating disease.

## MicroRNA biogenesis

**Fig. 1:**
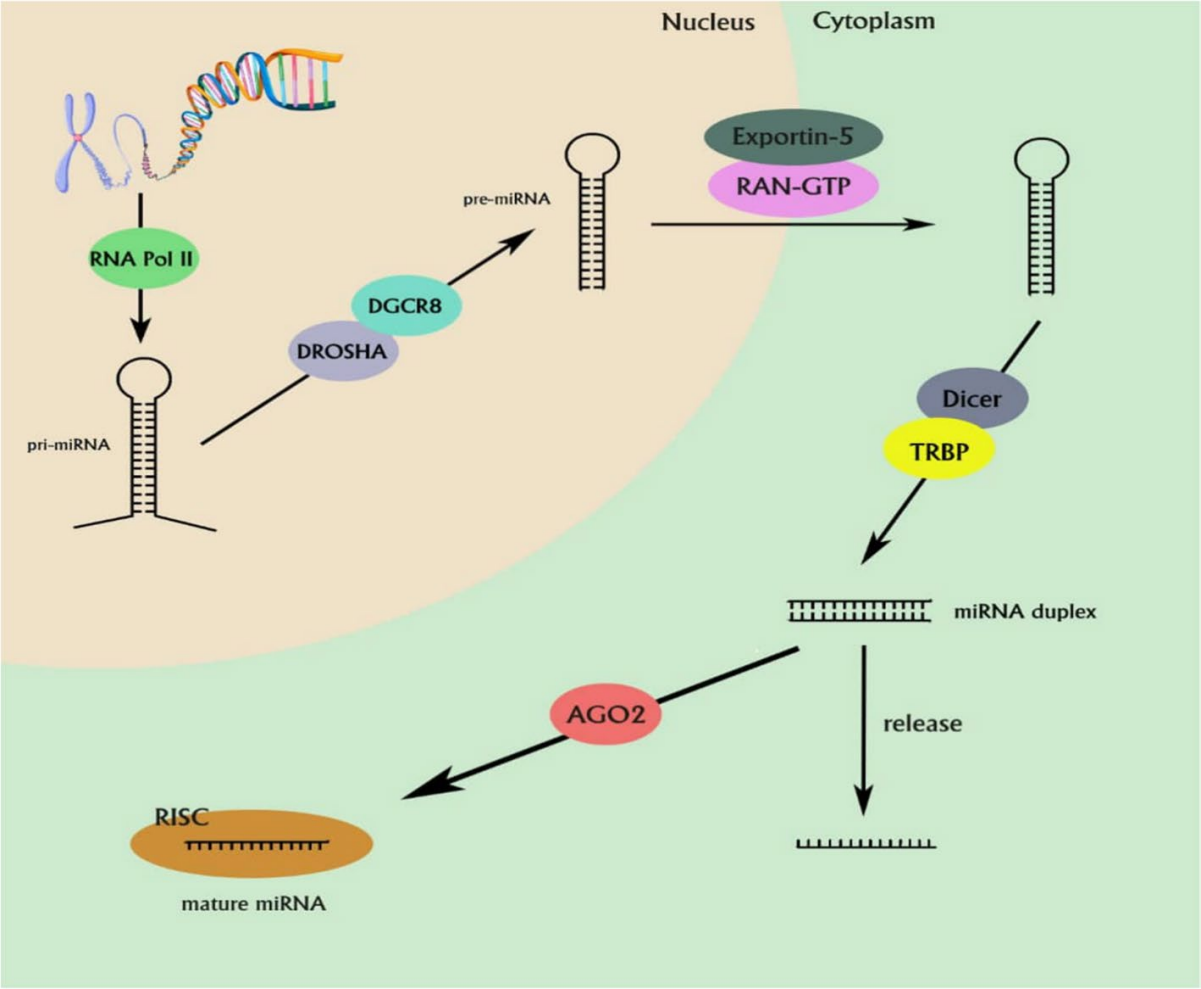
The miRNAs biogenesis pathway including several sequential stages, primarily initiated by the RNA polymerase II-mediated miRNA transcription (step 1), which is processed by the enzyme DROSHA and DGCR8 within the nucleus (step 2). The resulting precursor miRNA is exported from the nucleus to the cytoplasm through exportin 5, where it is processed subsequently by the Dicer enzyme to form mature miRNAs (step 3–4). Finally, the mature miRNAs are incorporated into the RISC with Ago proteins (step 6), where they regulate gene expression. RISC: RNA-Induced Silencing Complex

miRNAs biogenesis flows from two different ways, which are nuclear and cytoplasmic processing. Firstly, in the nucleus, RNA polymerase II binds to DNA and transcribes primary miRNA (pri-miRNA) which undergoes a second process by a microprocessor complex comprising RNA III enzyme DROSA and Di George critical region 8 (DGCR8) and a double-strand RNA binding protein to form precursor miRNA (pre-miRNA) with a stem-loop structure [11]. Subsequently, the pre-miRNA is exported to the cytoplasm through the (RAN-GTP)-dependent transporter exportin-5 [12]. Then the cytoplasmic RNA III enzyme, Dicer ribonuclease, complexed with TAR RNA binding protein, binds to the pre-miRNA hairpin and generates the double-stranded precursor miRNA (ds-miRNA) which is unstable. One strand is incorporated into the cytoplasmic RISC (RNA-Induced Silencing Complex) and the other strand (passenger strand) is removed (Fig. 1) [13].

## MicroRNA mechanisms of action

Both plants and animals contain miRNA molecules. They typically contain approximately 22 nucleotides and regulate gene expression by recognizing and binding to the complementary 3'untranslated region (UTR) of target mRNA at the post-transcriptional level. This binding can lead to either mRNA degradation or inhibition of translation of the target gene [14, 15]. miRNAs play a vital role in numerous biological processes, including proliferation, tissue differentiation, apoptosis, neural development, and pathogenesis [14, 16, 17]. In addition, recent data has inferred that miRNAs substantially participate in the initiation, progression, and development of cancers via either tumorigenesis or suppression of cancer cells. Dysregulation of miRNAs has been identified in many types of cancers, including glioma [18–20].

Identification of the underlying pathogenic mechanisms of miRNAs in cancer biology is critical for developing effective diagnostic and therapeutic strategies. There is promising evidence suggesting that miRNAs have the potential to act as either prognostic and diagnostic biomarkers or therapeutic (theranostic) targets in a variety of cancers, including glioma. By elucidating the role of miRNAs in glioma development and progression, we can gain a better understanding of the disease and develop more effective treatment options for patients [21–23].

## miRNAs in glioma

MiRNAs play a crucial role in the initiation and progression of many cancer types, including glioma. These non-coding RNAs serve as prognostic and diagnostic biomarkers for tumors due to their involvement in diverse biological processes, such as cell differentiation, apoptosis, metastasis, and tumorigenesis [24]. There are abundant miRNAs in human body fluids including plasma, urine, saliva, and cerebrospinal fluid. Deregulation in miRNA processing and release is associated with the onset of various cancers [25]. miRNAs can modulate many target mRNA molecules in the same pathway [26], acting as either tumor suppressors or oncogenes by negative regulation of target mRNAs, causing degradation or translational inhibition [27, 28].

**Fig. 2:**
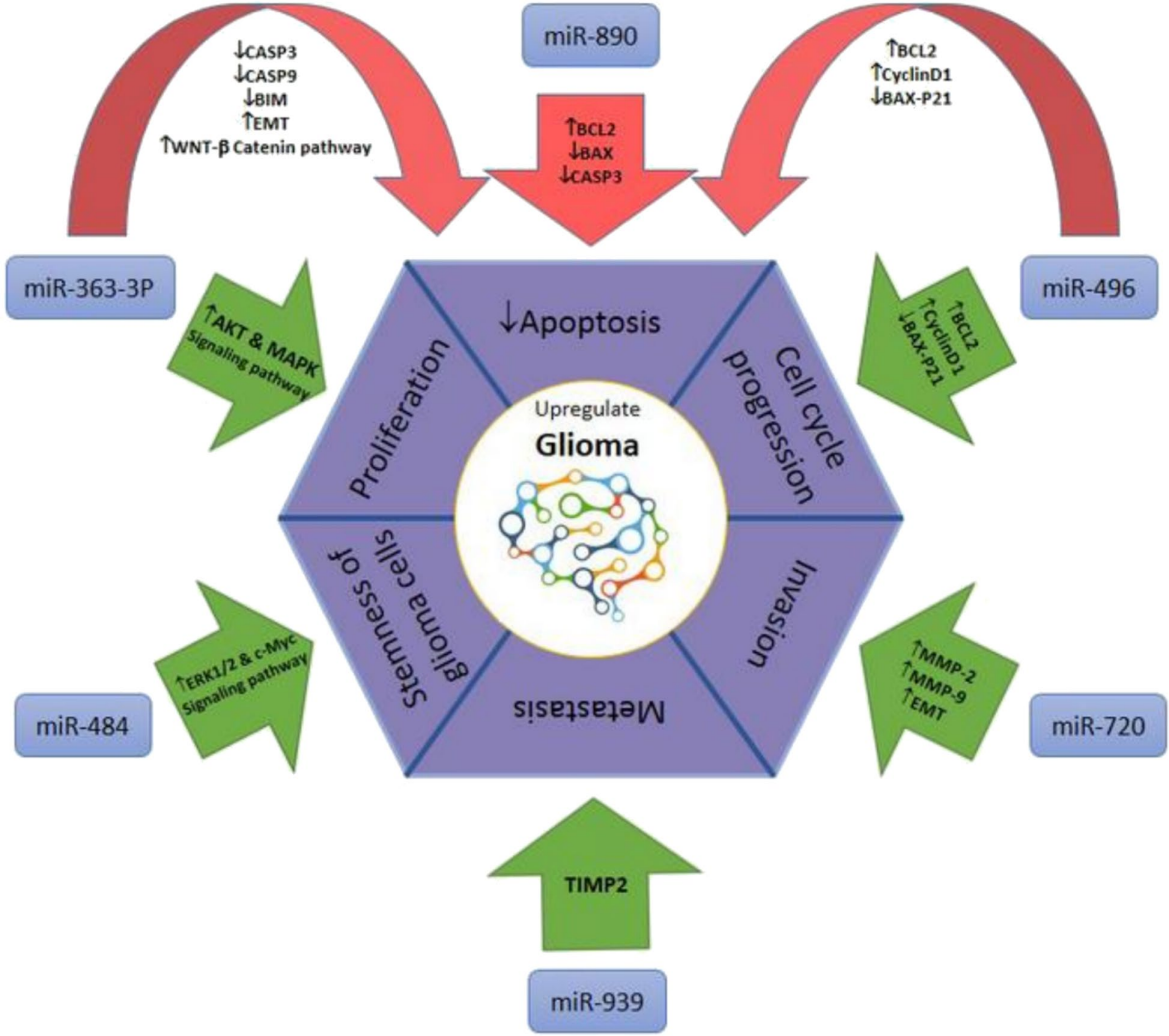
Mechanisms of action of oncogenic miRNAs including miR-484, miR-939, miR-720, miR-496, miR-363-3P, and miR-890 in the upregulation of glioma via various cellular pathways; their oncogenic mechanisms result in cell cycle progression, cell proliferation, stemness of glioma cells, invasion, metastasis, and inhibition of apoptosis

Currently, surgery combined with chemotherapy and radiotherapy is the standard treatment for glioma (9). However, this treatment is insufficient, and the mean survival time is less than two years. Hence, we require identifying molecular biomarkers for novel therapeutic strategies. Two strategies are involved in miRNA in glioma treatment: upregulating tumor suppressor miRNAs, also designated as miRNA mimics, and downregulating tumor inducer miRNAs [29, 30] (Table 1 and Fig. 2). miRNAs can regulate a variety of mRNAs, and each mRNA may also be regulated by a variety of miRNAs [31]. Thus, miRNA therapy in glioma is based on the overexpression of tumor suppressive miRNAs and inhibiting the activity of oncogenic miRNAs. In addition, miRNA plays a crucial role in the regulation of those genes responding to chemo and radiotherapy. Some miRNAs are responsible for TMZ (temozolomide) resistance in cells, and some increase glioma cell sensitivity to TMZ chemotherapy [32].

**Table 1:** Various miRNAs and their mechanisms in the regulation of glioma

miRNA name	Mechanism	Reference
miR363-3P	↑Wnt/β-catenin, EMT, and AKT-MAPK1 ↓CASP3, CASP9, BIM, and PDHB	[31]
	↑MMP2, MMP9, and EMT	
miR-720	↑ ERK1/2 and C-Myc signaling pathway	[32, 33]
miR-484	↑Proliferation and Metastasis	
miR-939-5P	↑ BCL2, and cyclin D1 ↓BAX and P21	[34]
miR-496	↑BCL2 ↓BAX and Caspase 3	[35]
	↑Wnt/β-catenin, EMT, and AKT-MAPK1 ↓CASP3, CASP9, BIM, and PDHB	
miR-890	↑MMP2, MMP9, and EMT	[36]
	↑ ERK1/2 and C-Myc signaling pathway

## miR363-3P

miR-363-3P has shown a crucial role in the progression of various cancers. In ovarian cancer, the actin cytoskeleton regulation via hsa-miR-363-3P-SPOCK2 axis was associated with advanced cancer stage [33]. The Dickkopf 3 targeting, increased cell viability, and migration have been associated with prostate cancer progress [34]. Inversely, miR-363-3P inhibited the bladder cancer cell proliferation and migration, while the overexpression of BTG2 reversed its inhibitory effect [35]. Additionally, miR-363-3P inhibited the growth of osteosarcoma (U2OS and MG63) cells via SOX-4 targeting [36]. The suppressor role of miR-363-3P in colorectal cancer has been exerted via SphK2 targeting [37]. miR-363-3P plays a significant role as an oncogene to promote cancer development of glioma via several signaling pathways. Its expression level is directly related to the glioma grade [38]. The expression of miR363-3P is upregulated in glioma, and thus it increases cell proliferation and viability and promotes glioma cells metastasis. miR363-3P expression rate is significantly higher in glioma tissue than in nearby normal brain tissue [39]. It is involved in various gene processes during glioma development. miR363-3P diminishes the expression of tumor suppressor CELF2 through the Wnt/β-catenin pathway and driving Epithelial–mesenchymal transition (EMT) or epithelial mesenchymal transition, thereby resulting in reduced cell death and increased metastasis. Treatment with miR363-3P inhibitor leads cells to cease the glioma cell cycle and inhibit cell growth [40]. miR363-3P suppresses apoptotic mRNAs, Caspase 3, Caspase 9, and Bim protein in CD133 + glioma stem cells, promoting dysregulation of apoptosis [41]. Interestingly, emerging evidence has demonstrated that miR363-3P, reversely targeting PDHB (Pyruvate Dehydrogenase B) has a tumor-suppressive function and reduces the expression of PDHB mRNA and protein levels [39]. Furthermore, miR363-3P upregulates glioma by directly targeting the 3′ UTR of GAP-43 and downregulates the tumor suppressor GAP-43 through activation in AKT and MAPK signaling pathways [38]. Additionally, Bi et al. demonstrated that HNF1A-AS1 is a lncRNA acting as a miR363-3P sponge and promoting glioma progress through the JNK signaling pathway to regulate the expression of MAP2K4 (mitogen-activated protein kinase 4) [42]. To reinforce the “double nature” of miR363-3P, it has been implicated in tumor quenching in colorectal cancer (CRC), lung cancer, and hepatocellular carcinoma (HCC) [37, 43, 44]. In contrast, it worked as an oncogene in cancers such as leukemia and gastric cancer [45, 46].

## miR-720

miR-720 has been participated in various cancers. For instance, it has been negatively associated with breast cancer (targeting TWIST1) [47], pancreatic cancer cells (cyclin D1 targeting) [48], while positively promotes glioma migration (TARSL2 or Threonyl-tRNA synthetase-like 2 expression control) [49]. miR-70 has an incredible association with cancer cell invasion and metastasis. TARSL2 is a protein involved in protein synthesis via catalyzing the binding of amino acids to tRNA [50]. miR-720 promotes glioma by directly targeting the 3′-UTR of TARSL2, resulting in the downregulation of TARSL2 expression, which is negatively correlated with miR-720 expression levels in glioma tissue. miR-720 has been shown to increase matrix metalloproteinase (MMP)−2 and MMP-9 expression and promote EMT, leading to increased proliferation and invasion in glioma. High expression levels of miR-720 are associated with poor survival rates [51, 52]. Additionally, Chen et al. have reported that glioma tissues contained higher miR-720 plasma levels compared to those of normal brain tissues, being correlated with glioma grades. Elevated miR-720 expression rate is associated with recurrence and adverse outcomes and may serve as a moderate diagnostic tumor biomarker with a sensitivity of 71.3% and specificity of 83.3% (24). In addition, miR-720 has played a role as an oncogene and prognostic tumor marker in various cancers, including CRC (increased serum levels), renal cell carcinoma (E-cadherin-αE-catenin regulation), and triple-negative breast cancer (TNBC) [47, 50, 52, 53]. Thus, miR-720 is an oncogene and a prognostic tumor marker in glioma diagnosis, and its inhibition may prolong survival rate in glioma patients.

## miR-484

Encoded by chromosome 16p13.11, miR-484 plays some role in various cancers. These cancers mainly include clear cell renal cell carcinoma (targeting lncRNA PCED1B-AS1 and ZWB1) [54], colorectal cancer (SW480 and HCT116 cells and targeting lncRNA PGM5-AS1, CD137L, ZFAS1, and LINC00239) [55], pancreatic ductal adenocarcinoma (lncRNA THAP9-AS1 and YAP) [56], cervical cancer tissue (targeting MMP14 HNF1A and EZH2 DNMT1) [57], breast carcinoma cell lines (targeting KLF4 and CDA) [58, 59], ovary (VEGFB VEGFR2) [60], and glioma (MAP2 and cMyc) [61] cells [55]. miR-484 is upregulated in NSCLC, thyroid carcinoma, prostate cancer, HCC, and glioma [62–65]. miR-484 initiates glioma progression through targeting of MAP2, cell proliferation, and apoptosis inhibition resulting in an increase in tumor size. miR-484 knockdown has resulted in MAP2 overexpression, diminished the interaction between growth factor receptor bound protein 2 (Grb2) and son of sevenless (Sos) and downregulated ERK1/2 and c-Myc signaling pathways. C-Myc regulates miR-484 and therefore augments the stemness of glioma stem cells. Patients with both miR-484 high expression and MAP-2 low expression levels are associated with a more difficult prognosis and poor overall survival. In addition, miR-484 expression levels are significantly increased by glioma grade. Both MAP2 and miR-484 are considered potential predictive factors for the diagnosis and treatment of glioma (33).

## miR-939-5p

miR-939-5p has been associated with various cancers. The miR-939-5p downregulation inhibited the pancreatic cancer cell invasion, viability, and migration by targeting Rho GTPase-activating protein 4 (ARHGAP4) [66]. TNBC cell progression modulation was associated with the interplay between lncRNA HEIH/miR-939-5p which precluded the NOS2 and NOS3 transcripts [67]. In addition, miR-939-5p sponging by lncRNA LINC00460 has resulted in CRC cell (HT29 and LOVO) metastasis promotion mediated by LIM kinase 2 (LIMK2) [68]. miR-939 targeting by lncRNA RP5-916L7.2 has been associated with advanced tumor stage, apoptosis inhibition, and cell proliferation in tongue squamous cell carcinoma (Tca-8113 cells) [69]. The expression levels of miR-939-5p are significantly upregulated in glioma and correlate with tumor size and WHO glioma grades. miR-939-5p overexpression has been exhibited in various types of cancers. For instance, in pancreatic cancer, miR-939-5p promotes tumor growth by targeting ARHGAP4 [66]. Similarly, in HCC, the overexpression of miR-939-5p negatively targets ESR1, leading to tumor progression [70]. In addition, miR-939-5p has been upregulated in lung cancer, where it promoted migration and invasion [71]. Taken together, these findings suggest that miR-939-5p may serve as a potential therapeutic target for cancer treatment. More relevantly, miR-939-5p acts as an inducer by targeting tissue inhibitors of metalloproteinase-2 (TIMP2), which results in increased proliferation, invasion, and metastasis of glioma cells. Notably, overexpression of miR-939-5p predicts a less favorable outcome in glioma patients [72]. Further investigations are warranted to elucidate the underlying mechanisms and to explore the clinical significance of miR-939 in cancer.

## miR-496

miR-496 plays a dual role in cancers. The CRC cell migration and EMT (Wnt pathway-mediated) have been related to the Ras-association domain family 6 (RASSF6) targeting [73]. The miR-496/DDIT4 axis regulation by NNT-AS1 has resulted in the proliferation and migration of PCa prostate cancer cells [74]. miRNA-496 has also developed gastric cancer via participation in non-coding RNA activated by DNA damage (NORAD)-interleukin-33 axis in the GC cell–CAFs co-culture model [75]. Additionally, its participation in the mTOR pathway has been related to CRC development [76]. The LYN and AKT targeting in gastric cancer has been associated with AGS cell proliferation inhibition [77]. miR-496 is highly expressed in glioma cells, being inversely correlated with the expression of HLA complex group 11 (HCG11). The upregulation of miR-496 and the downregulation of HCG11 are associated with lower survival rates. Interestingly, HCG11 overexpression is related to apoptosis and cell cycle arrest through reducing Bcl2 and cyclin D1 and increasing Bax and p21 expression levels. The underlying mechanism of the HCG11 downregulation in glioma is the inhibition of FOXP1-driven transcription. HCG11 suppressed glioma tumorigenesis in vitro and in vivo by competitive binding to the 3′UTR of miR-496 with cytoplasmic polyadenylation element binding protein 3 (CPEB3) and blocking the wnt/β-catenin pathway. Therefore, the HCG11/miR-496/CPEB3 axis may be a potential prognostic indicator in the early diagnosis and treatment of glioma [78]. Additionally, miR-496 has been implicated in the progression of several types of cancers, such as CRC, prostate cancer, and nasopharyngeal carcinoma [73, 79, 80].

## miR-890

miR-890 may have an inhibitory or promoting effect in cancers. It has targeted CD147 in TNBC and thus induces apoptosis and inhibits cell proliferation and invasion, and also MMP expression inhibition and Caspase-3 cleavage [81]. Moreover, the upregulation of ELK3 mediated by miR-890 sponged by LINC00662 has resulted in melanoma cells (A375 and SK-MEL-1) proliferation, migration, and invasion [82]. It has been unraveled that miR-890 downregulation by lncRNA SNHG3 was associated with lung adenocarcinoma initiation and development [83]. miR-890 is another factor with overexpression in glioma cells compared to that of normal brain cells. Using GSE86202 and GSE92322 data sets, hsa_circ_0008225 has acted as a competing endogenous RNA (ceRNA) by silencing miR-890 to regulate the expression of its target, zinc finger MYND-type containing 11 (ZMYND11), thereby suppressing glioma progression. Overexpression of hsa_circ_0008225 precluded cell growth and invasion in glioma tissue and also induced cell apoptosis through an increase in BAX pro-apoptotic protein and caspase3 and a reduction in BCL2 anti-apoptotic protein expression levels. Furthermore, low expression levels of hsa_circ_0008225 are associated with cancer recurrence and poor patient survival. These findings suggest that hsa_circ_0008225 acts as an anti-oncogene circRNA in glioma suppression by regulating the hsa_circ_0008225/miR-890/ZMYND1 axis [84]. miR-890 is correlated with a large number of cancers; for instance, lncRNA SNHG3 promoted lung adenocarcinoma by modulating miR-890 [83]. LINC00662 enhances cell proliferation and promotes melanoma progression through the miR-890/ELK3 pathway [82]. Conversely, according to existing data, miR-890 downregulation is associated with TNBC repression via targeting CD147 [81].

## Clinical translation of miRNAs

miRNAs have been detected in various samples such as serum, serum exosomes, blood or peripheral blood, plasma or plasma exosomes, CSF, and tissues functioning as biomarkers [85, 86]. For example, miR 362-3p and miR 363-3p have been detected in the serum of patients with adult-type glioma [87].

## Challenges in the delivery of miRNAs

The presence of blood–brain barrier (BBB) restricts the passage of miRNAs from the bloodstream into the brain [88]. In addition, blood and extracellular environments contain enzymes that affect miRNAs stability [89]. The achievement of specific miRNA targets is also challenging where their nonspecific distributions may exert off-target toxicity [90]. Their cellular uptake by glioma cells is not also convenient necessitating appropriate delivery vectors [91]. Moreover, miRNA vectors are associated with immunologic reactions and toxicity which complicate clinical translation [90, 92–94]. Gliomas high heterogeneity also impedes miRNAs delivery and distribution. miRNAs production and their scaling up is also a difficult process [95, 96].

## Future insights in miRNAs therapy in glioma

Personalized miRNA therapeutics is a promising approach considering individual tumor miRNA profiles to increase the therapeutic efficacy and mitigate off-target effects [97]. Novel delivery systems, including nanocarrier-based delivery, viral vectors, and local delivery approaches, also improve therapy outcomes [98]. The application of combination therapies, such as assessment of synergistic effects and applying multiple targets, also enhances therapeutic effects [99, 100]. The understanding of miRNA networks using systems biology and their contributing roles in resistance also increases the efficiency of therapies [101, 102].

## Conclusion

Recent studies have revealed the crucial association of miRNAs as innovative and remarkable diagnostic, prognostic, and therapeutic targets in suppressing glioma tumorigenesis. The use of miRNAs in combination with surgery and chemotherapy will be a potential therapeutic approach against glioma. Therefore, miRNAs have a significant signature in cancer progression, and advancement in the understanding of miRNA processes is crucial. This will contribute to triumph in the early diagnosis of cancers and improvement of survival time. The present study identified six inducer miRNAs, listed as miR-363-3p, miR-720, miR-484, miR-890, miR-496, and miR-939-5p which can promote glioma stem cells through post-transcriptional degradation or translational inhibition [40, 72, 78, 84, 103, 104]. These miRNAs have been upregulated in glioma and acted as oncogenes. The high expression levels of oncogenic miRNAs in glioma are associated with cell proliferation, tumor differentiation, invasion, metastasis, and angiogenesis [105]. Albeit miRNA-based diagnosis and treatment of glioma have been considered a promising strategy for future developments, further studies are warranted to translate these molecular mechanisms into clinical practice as a precision medicine intervention.

## Data Availability

All required data/in any will be provided by the corresponding author.

## Abbreviations

miRNA: MicroRNA
CNS: Central nervous system
WHO: World Health Organization
GBM: Glioblastoma multiforme
DGCR8: Di George critical region 8
RISC: RNA-Induced Silencing Complex
UTR: Untranslated region
TMZ: Temozolomide
NSCLC: Non-small cell lung cancer
EMT: Epithelial–mesenchymal transition
PDHB: Pyruvate Dehydrogenase B
MAP2K4: Mitogen-activated protein kinase 4
CRC: Colorectal cancer
HCC: Hepatocellular carcinoma
TARSL2: Threonyl-tRNA synthetase-like 2
MMP: Matrix metalloproteinase
TNBC: Triple-negative breast cancer
Grb2: Growth factor receptor bound protein 2
Sos: Son of sevenless
ARHGAP4: Rho GTPase-activating protein 4
LIMK2: LIM kinase 2
TIMP2: Metalloproteinase-2
RASSF6: Ras-association domain family 6
HCG11: HLA complex group 11
CPEB3: Cytoplasmic polyadenylation element binding protein 3
ceRNA: Competing endogenous RNA
ZMYND11: Zinc finger MYND-type containing 11
